# Exploring Student and Faculty Insights on Ultrasound Education for Final-Year Medical Students: A Qualitative Study

**DOI:** 10.2147/AMEP.S621984

**Published:** 2026-09-13

**Authors:** Khalid Bashir, Amani A Al-Rajhi, Munirah Altaissan, Asma Syed, Maha Desouki, Najlaa Al-Meraikhi, Tanya Kane

**Affiliations:** 1 Emergency Department, Hamad General Hospital, Doha, Qatar; 2 Clinical Department, College of Medicine, QU Health, Qatar University, Doha, Qatar; 3 Department of Population Medicine, College of Medicine, QU Health, Qatar University, Doha, Qatar

**Keywords:** ultrasound, undergraduate medical education, qualitative research, curriculum development, Qatar

## Abstract

**Background:**

Ultrasound is an important bedside valuable tool, but its integration into undergraduate medical education remains variable, particularly in the Middle East and North Africa (MENA) region. At Qatar University (QU) College of Medicine, ultrasound is introduced during the preclinical years, while clinical exposure remains largely opportunistic. This study explored student and faculty perspectives on the strengths and limitations of the ultrasound curriculum at QU.

**Methods:**

A qualitative study used focus groups with final-year medical students (n=21) and semi-structured interviews with faculty involved in ultrasound training (n=15). Data were analyzed using a triangulated thematic approach combining independent manual coding with AI-assisted analysis. Final themes were refined through researcher consensus.

**Results:**

The main finding was a translational gap between early technical proficiency and the ability to apply ultrasound findings to clinical reasoning and decision-making. Four student themes and five faculty themes emerged. Early preclinical exposure was valued for developing technical skills and confidence. However, clinical clerkship exposure was often opportunistic and lacked structured integration. Students reported confidence in image acquisition but limited exposure to pathology and clinical application. Faculty similarly recognized strong technical skills but identified weaknesses in interpretation, diagnostic reasoning, and clinical integration. Both groups supported a longitudinal, spiral curriculum with structured exposure to abnormal findings.

**Conclusion:**

This study identifies a clear gap between technical ultrasound skills and their application in clinical practice. Bridging this gap requires moving from isolated preclinical teaching toward a longitudinal, clinically integrated curriculum that supports interpretation and clinical reasoning. These findings may inform ultrasound curriculum development in the MENA region and similar settings.

## Background

The advent of portable ultrasound technology has significantly transformed clinical practice, extending its application beyond radiology into point-of-care settings for rapid diagnostic assessment.^1^,^2^ This shift has been accompanied by strong endorsements from professional bodies advocating for the integration of ultrasound education into undergraduate medical curricula.^3–5^ Traditionally, anatomy and clinical skills teaching has relied on cadaveric dissection and textbook-based learning, approaches that may limit students’ understanding of three-dimensional anatomy and dynamic physiological processes.^6^,^7^ In contrast, ultrasound provides real-time visualization of living anatomy, serving as an effective bridge between foundational sciences and bedside clinical assessment.^7–9^ Evidence demonstrates that ultrasound enhances spatial understanding, improves correlation with physical examination findings, and supports the development of procedural skills.^10–13^

Globally, medical schools are increasingly incorporating ultrasound into undergraduate curricula, often through progressive models that transition from foundational anatomical learning to clinically indicated applications.^14–17^ Despite this progress, guidance for undergraduate ultrasound education remains insufficiently defined. Existing frameworks are frequently extrapolated from postgraduate training and lack clearly articulated competencies tailored to medical students.^5^ In the Middle East and North Africa (MENA) region, implementation is further constrained by financial, logistical, and resource-related challenges.^18^

At Qatar University (QU) College of Medicine, ultrasound is introduced during the second (preclinical) year of a six-year undergraduate program. Training follows a system-based approach, integrating theoretical instruction with hands-on practice using point-of-care ultrasound (POCUS) on simulated patients, and is assessed through an Objective Structured Clinical Examination (OSCE). However, during clinical clerkships, exposure to ultrasound is largely opportunistic, with limited structured integration into clinical learning.

The literature on undergraduate ultrasound education remains fragmented across different study designs and curricular contexts. Some studies have examined student perceptions and self-reported confidence,^19^ whereas others have evaluated short training interventions, focused ultrasound modules, simulation approaches, institutional implementation, or anatomy-focused applications.^20–25^ These studies provide useful evidence regarding feasibility, learner experience, and acquisition of basic ultrasound skills, but they do not fully explain how early technical training is carried forward into clinical reasoning during later clerkships. Consequently, several gaps remain, including limited studies integrating student and faculty perspectives within the same undergraduate program, relatively limited representation from the MENA region, and insufficient stakeholder-informed guidance on how to bridge preclinical training with clinical application.

This fragmentation is important because different strands of the literature illuminate different parts of the educational pathway. Student-focused work has described perceptions, knowledge, confidence, and future expectations.^19^ Other studies have demonstrated gains from short courses or focused training,^20^,^21^ surveyed institutional approaches to undergraduate ultrasound education,^22^ or evaluated specific educational applications and training models.^23–25^ Longitudinal curricular examples have also been described, particularly outside the MENA region.^14–17^ However, less attention has been paid to the transition from acquiring images in controlled teaching settings to interpreting findings and integrating them into clinical decisions during authentic patient care. By triangulating student and faculty accounts within a single undergraduate program, the present study addresses this gap and offers a stakeholder-informed account of why the transition from technical skill to clinical application may remain incomplete.

A competency-based educational framework is useful for interpreting this transition. Miller’s Pyramid^26^ distinguishes progressive levels of clinical competence—“knows”, “knows how”, “shows how”, and “does”—and has been widely applied to characterize the gap between demonstrating a skill under controlled conditions and autonomously applying it in authentic patient care. Undergraduate ultrasound curricula, including OSCE-based assessment, are structured predominantly around the “shows how” level: learners demonstrate technique on standardized patients or phantoms in a supervised setting. However, the “does” level—selecting, performing, interpreting, and integrating ultrasound findings to answer a genuine clinical question—requires a different kind of learning experience. Constructs such as deliberate practice,^27^ which emphasizes repeated, feedback-rich engagement with progressively challenging tasks, and cognitive apprenticeship,^28^ in which expert reasoning processes are made explicit and gradually transferred to the learner, suggest that this progression cannot be achieved through technical instruction alone. These frameworks provide the conceptual lens through which this study interprets the disconnect between technical skill acquisition and clinical application explored below.

Therefore, this study aims to explore the perspectives of both medical students and faculty at QU regarding the current ultrasound curriculum. By integrating these perspectives, the study seeks to evaluate strengths, identify systemic challenges, and generate evidence-based recommendations to support the development of a structured, longitudinal, and clinically integrated ultrasound education framework.

## Methods

### Study Design

This exploratory qualitative study investigated experiences and perceptions of ultrasound education at QU College of Medicine from the dual perspectives of final-year medical students and teaching faculty. Data were collected using focus group discussions with students (Appendix A, provided as a separate supplementary file) and semi-structured interviews with faculty members (Appendix B, provided as a separate supplementary file). The study adhered to the *COnsolidated Criteria for REporting Qualitative Research* (COREQ) guidelines.

### Participants and Recruitment

A convenience sampling strategy was employed to capture a broad range of perspectives within this single-institution study. All eligible final-year medical students (n=69) and faculty members involved in delivering the undergraduate ultrasound curriculum (n=19) were invited via Email and messaging platforms. Information sheets outlined the voluntary and non-evaluative nature of participation.

Inclusion criteria for students were enrolment as a final-year medical student at QU and provision of informed consent. Faculty inclusion criteria included involvement in teaching or supervising undergraduate ultrasound training and provision of informed consent. Emergency medicine faculty were specifically targeted, as they deliver the preclinical ultrasound curriculum and supervise students during final-year rotations.

A total of 21 students and 15 faculty members consented to participate. Data saturation was achieved when no new themes emerged across two consecutive student focus groups and three consecutive faculty interviews. All interviewers (AA, MA, NA) were certified in human subjects research through the Collaborative Institutional Training Initiative (CITI), and interviews were conducted under the supervision of senior researchers (KB, TK).

Of the 69 eligible students and 19 eligible faculty invited, 21 students (30%) and 15 faculty (79%) ultimately consented to participate. Reasons for non-participation were not systematically recorded; however, scheduling conflicts and clinical workload were cited informally by several eligible students. As with any convenience sample recruited through open invitation, participants who volunteered may differ systematically from non-participants in their engagement with, or attitudes toward, ultrasound training—for example, more motivated learners or more critically engaged faculty may have been over-represented. This potential selection bias, and its implications for the transferability of the findings, is discussed further in Strengths and Limitations.

### Study Setting: Ultrasound Curriculum at QU

Ultrasound education at QU is introduced during the second (preclinical) year within a six-year undergraduate medical program. The curriculum follows a system-based approach, beginning with foundational concepts (eg, knobology, safety, and probe handling) and progressing to organ-specific modules aligned with the broader curriculum (eg, cardiac views during the cardiovascular block).

Teaching is delivered using a structured *show–practice–feedback* model in small groups, incorporating both simulated patients and training phantoms. The total scheduled teaching time is approximately 26–30 hours. Student competency is assessed through formative and summative Objective Structured Clinical Examinations (OSCEs), primarily focused on image acquisition of normal anatomy. In contrast, exposure during clinical clerkships is largely opportunistic and lacks formal curricular integration.

### Ethical Considerations

Ethical approval was obtained from the Qatar University Institutional Review Board (QU-IRB 334/2024-EA) under expedited review in accordance with Qatar Ministry of Public Health regulations. The study was classified as minimal risk. Written informed consent was obtained from all participants. Confidentiality was maintained through de-identification of transcripts and secure data storage. Participants consented to the use of anonymized quotations in dissemination.

### Data Collection

Interview guides were pilot-tested with three students to ensure clarity and relevance. Minor revisions were made to two student questions; no changes were required for the faculty guide.

Data were collected through 10 student focus groups (2–3 participants per group; duration 25–40 minutes) and 15 individual faculty interviews (duration 10–60 minutes). Variation in interview duration reflected participant availability; shorter interviews still yielded meaningful insights, while longer interviews allowed deeper exploration. Data saturation was achieved across both groups. All interviews were conducted in English. Most sessions were held via Microsoft Teams, with seven faculty interviews conducted face-to-face.

### Data Analysis

Data were analyzed using thematic analysis guided by Braun and Clarke’s six-phase framework. To enhance analytical rigor, a triangulated approach was employed, combining independent manual coding with AI-assisted preliminary coding, followed by consensus-based theme development.

Author KB conducted independent inductive coding across all transcripts, generating initial codes and a preliminary thematic framework. In parallel, Authors AA and MA used a large language model (ChatGPT, GPT-4o) to generate preliminary descriptive codes from anonymized transcript excerpts. AI outputs were treated as exploratory and were critically reviewed, refined, or discarded by the researchers. All three analysts independently developed initial thematic maps. These were subsequently compared and refined through iterative consensus meetings, with discrepancies resolved through discussion and repeated reference to the raw data. Final themes were agreed collaboratively, ensuring that interpretations remained grounded in participant narratives.

The triangulated analytical workflow is summarized in Table 1 (placed at the end of the manuscript, following the reference list).

**Table 1:** Overview of the Triangulated Thematic Analysis Process Combining Manual and AI-Assisted Coding

Stage	Manual Inductive Coding (KB)	AI-Assisted Coding with Human Review (AA, MA)
Data Source	Complete de-identified transcripts from 10 student focus groups and 15 faculty interviews (25 transcripts; 36 participants)	Same dataset: 25 de-identified transcripts representing 36 participants
Initial Coding	Inductive coding performed manually; identification of meaningful units and development of preliminary codebook	GPT-4o used to generate preliminary descriptive codes from anonymized text; outputs critically reviewed, refined, or discarded by researchers
Theme Development	Development of initial thematic framework and thematic map based on patterns across the dataset	AI-assisted descriptive codes were reviewed by researchers and considered alongside manual coding during development of a provisional thematic framework
Consensus Process	Iterative comparison of coding frameworks across analysts; discrepancies resolved through discussion and reference to raw data	Integrated into consensus discussions; AI-derived codes accepted, modified, or rejected based on team agreement
Final Output	Agreed thematic framework: 4 student themes and 5 faculty themes	AI contribution limited to preliminary coding; all final themes derived through human consensus

**Abbreviations**: AI, artificial intelligence; GPT-4o (OpenAI).

### Reflexivity

The research team comprised faculty members involved in ultrasound teaching (KB) and medical students who had previously completed the ultrasound curriculum (AA, MA). This dual positioning introduced the potential for social desirability bias during data collection and interpretive bias during analysis. To address this, student researchers (AA, MA) led the focus group discussions, creating a more open environment for participants and reducing perceived power imbalances. Faculty involvement in interviews was carefully managed to minimize hierarchical influence.

During data analysis, the research team adopted a reflexive approach. Researchers explicitly documented their assumptions prior to coding and engaged in ongoing discussions to challenge interpretations. Particular attention was given to identifying disconfirming evidence to avoid reinforcing pre-existing views. All themes were repeatedly cross-checked against the original transcripts to ensure that findings remained grounded in participants’ perspectives.

### Use of Artificial Intelligence in Analysis

A large language model (GPT-4o, OpenAI) was used solely to assist with preliminary descriptive coding of anonymized transcripts. All identifying information was removed prior to use, and no sensitive data were shared.

Anonymised transcript excerpts were provided to the model using the following structured prompt: “Analyse this anonymised qualitative interview text from a medical education study. Identify recurring concepts, themes, or patterns within this excerpt. For each concept you identify, generate a short descriptive label without applying a pre-specified coding framework. Group related concepts into provisional categories where appropriate. Do not interpret or infer meaning beyond what is stated in the text”. Transcripts were processed in batches of 3–5 excerpts per session, depending on transcript length. Every AI-generated code and category was independently cross-checked by AA and MA against the corresponding source transcript. Codes that could not be substantiated in the underlying text, that duplicated existing manual codes, or that appeared to over-interpret participant meaning were discarded. Retained AI-generated codes were treated as a supplementary, exploratory starting point rather than a final coding scheme, and were integrated into the broader human consensus process described in Data Analysis alongside the independent manual coding performed by KB.

AI outputs were limited to preliminary descriptive coding and did not independently determine theme development, interpretation, or manuscript writing. All final themes, coding decisions, and analytical interpretations were determined by the research team through consensus.

## Results

Across both student and faculty datasets, the central and most consistent finding was a translational gap between technical proficiency in ultrasound and the ability to integrate ultrasound findings into clinical reasoning and decision-making. This gap, elaborated in the themes below and revisited as the central focus of the Discussion, represents the principal contribution of this study.

Thematic analysis identified four key themes from student data and five themes from faculty data (Tables 2 and 3, placed at the end of the manuscript, following the reference list).

**Table 2:** Student Perspectives on Ultrasound Education (n = 21)

Theme	Subtheme	Example Quote	Students (n)
**Privileged yet Disconnected Experience**	Early preclinical exposure	“My first exposure was during my second year… we were actually doing something a doctor does”.	21
	Inconsistent clinical exposure	“In clinical years it depended entirely on the rotation… you mostly just observe”.	21
**Foundation for Confidence, Not Diagnostics**	Anatomical understanding	“A textbook diagram is static, but this was alive”.	18
	Technical competence	“I knew the probes… even if I wasn’t making the diagnosis”.	15
**Systemic Challenges & Self-Directed Learning**	Technical/practical challenges	“Each machine was different… you’d finally get comfortable, then have to start over”.	13
	Peer-assisted learning	“We stayed after classes… practicing together”.	13
**Vision for Integrated Curriculum**	Structured clinical training	“Each rotation should have a dedicated ultrasound objective… not a bonus”.	17
	Limited exposure to pathology	“I can find a liver, but not early cirrhosis”.	9
	Assessment preferences	“The OSCE is too rushed”.	11

**Note**: The “Students (n)” column reflects the number of participants expressing each view.

**Table 3:** Faculty Perspectives on Ultrasound Education (n = 15)

Theme	Subtheme	Example Quote	Faculty (n)
**Strong Technical Foundation, Immature Clinical Integration**	Technical skill vs clinical integration	“They are very good at identifying where to place the probe”.	13
	Diagnostic gap	“The skill of ultrasound is one thing, but clinical experience… should be used together”.	2
**Competence in Normality, Deficiency in Abnormality**	Normal anatomy proficiency	“They are good at imaging anatomy… but not true pathology”.	12
**Variable Clinical Correlation**	Integration with patient history	“Some students just concentrate on getting the image—no correlation with the patient”.	4
**Early Exposure Enhances Overall Preparedness**	Improved readiness	“Residents from QU pick up advanced skills much faster”.	14
**Need for Pathological Exposure & Enhanced Assessment**	Structured pathology exposure	“Simulated mannequins with positive findings… or real patients”.	7
	Assessment beyond OSCE	“In DOPS, we can add image acquisition, interpretation, and clinical correlation”.	2

**Note**: The “Faculty (n)” column reflects the number of participants expressing each view.

### Student Perspectives (n = 21)

#### Privileged yet Disconnected Experience

All participants described early preclinical ultrasound exposure as a highly positive and motivating experience. One student noted, “My first exposure was during my second year… we were actually doing something a doctor does”. Several highlighted this as a distinguishing feature compared to peers in other institutions.

However, this positive experience contrasted sharply with clinical clerkships. All students reported that ultrasound exposure during clinical years was inconsistent and largely dependent on individual rotations. As one participant explained, “In clinical years it depended entirely on the rotation… you mostly just observe”. Consequently, a previously structured and mandatory skill became optional and variably reinforced.

#### Foundation for Confidence, Not Diagnostics

Students consistently reported that ultrasound enhanced their understanding of anatomy (n = 18) and, to a lesser extent, physiology (n = 9). As one participant described, “A textbook diagram is static, but this was alive”.

Technical confidence was also evident, with many students reporting competence in probe handling and basic image acquisition (n = 15). However, a clear gap emerged between technical proficiency and diagnostic capability. While students recognized the clinical utility of ultrasound (n = 13), their experiences were largely observational, limiting their ability to integrate findings into clinical decision-making. In essence, training enabled them to “see” structures but not yet to “interpret” or “decide”.

No student accounts substantially contradicted this overall pattern. While a minority of students (n = 3) expressed moderate confidence in applying ultrasound findings clinically, they explicitly attributed this to individual initiative and external mentorship rather than to structured curriculum design. As one student explained: “I had a good ED mentor who helped me gain confidence rather than college teaching”. This account, while representing a minority perspective, paradoxically reinforces the dominant finding: where clinical confidence developed, it did so despite, not because of, the formal curriculum—through self-directed efforts and supportive mentorship rather than through structured curricular experiences. Across the dataset, the overwhelming narrative remained one of technical confidence without corresponding diagnostic application.

#### Systemic Challenges and Self-Directed Learning

Students identified several systemic challenges, particularly related to technical variability across ultrasound machines (n = 13). As one participant noted, “Each machine was different… you’d finally get comfortable, then have to start over”. Additional challenges included patient-related factors, such as body habitus, which affected image quality.

In response to limited structured clinical exposure, students increasingly relied on self-directed learning strategies. Peer-assisted learning emerged as a key approach (n = 13), with students organizing informal practice sessions. “We stayed after classes… practicing together”, one participant reported. Independent practice was also common (n = 11), highlighting a proactive approach to skill development despite curricular limitations.

#### Vision for an Integrated Curriculum

Students strongly advocated for a more structured and longitudinal integration of ultrasound into clinical training (n = 17). One participant suggested, “Each rotation should have a dedicated ultrasound objective… not a bonus”.

A major limitation identified was the predominant focus on normal anatomy, with insufficient exposure to pathological findings (n = 9). As one student stated, “I can find a liver, but not early cirrhosis”. Participants also recommended expanding the curriculum to include additional applications, such as musculoskeletal, thyroid, and vascular ultrasound (n = 10).

Regarding assessment, several students (n = 11) expressed concerns about the current OSCE format, describing it as time-pressured and limited in evaluating clinical application. A preference for more formative, practice-oriented assessment methods was noted.

### Faculty Perspectives (n = 15)

#### Strong Technical Foundation, Immature Clinical Integration

Faculty consistently praised students’ technical proficiency in ultrasound (n = 13), particularly in probe positioning and image acquisition. As one faculty member noted, “They are very good at identifying where to place the probe”.

However, this technical competence was not matched by equivalent diagnostic ability. Faculty emphasized the importance of integrating ultrasound findings with clinical reasoning, which remained underdeveloped in some students (n = 2). One participant explained, “The skill of ultrasound is one thing, but clinical experience… should be used together”.

No faculty accounts substantially diverged from this overall pattern. One faculty member suggested that the observed gap may partly reflect individual differences in student motivation and initiative rather than curricular structure alone, commenting that “they are as good as residents with good knowledge and confidence in using ultrasound”. However, the majority view across the dataset was that the primary driver of the technical–clinical gap was the absence of structured, supervised clinical exposure to pathological findings and integrated diagnostic reasoning during clerkships.

#### Competence in Normality, Deficiency in Abnormality

Faculty observations closely aligned with student perspectives. While students demonstrated strong competence in imaging normal anatomy (n = 12), they showed limited ability to recognize and interpret pathological findings. As one faculty member stated, “They are good at imaging anatomy… but not true pathology”. This gap was largely attributed to training predominantly conducted on healthy individuals.

#### Variable Clinical Correlation

Faculty reported variability in students’ ability to integrate ultrasound findings into clinical context. In straightforward scenarios, such as trauma assessments, students performed well (n = 9). However, some participants (n = 4) observed that students prioritized image acquisition over clinical reasoning. As noted, “Some students just concentrate on getting the image—no correlation with the patient”.

#### Early Exposure Enhances Overall Preparedness

Despite identified gaps, faculty strongly valued early ultrasound training (n = 14). Many reported that graduates from QU demonstrated enhanced readiness and faster skill acquisition during postgraduate training. One faculty member commented, “Residents from QU pick up advanced skills much faster”.

#### Need for Pathological Exposure and Enhanced Assessment

Faculty emphasized the need for structured exposure to pathological findings (n = 7), suggesting the use of simulation-based training and real patient encounters. One participant recommended, “Simulated mannequins with positive findings… or real patients”.

In terms of assessment, some faculty (n = 2) advocated for workplace-based assessments such as Direct Observation of Procedural Skills (DOPS), which would allow evaluation of image acquisition, interpretation, and clinical integration. As one noted, “In DOPS, we can add image acquisition, interpretation, and clinical correlation”.

## Discussion

This dual-perspective study provides a stakeholder-informed evaluation of an undergraduate ultrasound curriculum at a medical school in Qatar, within the Gulf Cooperation Council (GCC) context. By integrating insights from final-year students and clinical faculty, we identify a fundamental translational gap in competency development: a disconnect between technical skill acquisition in preclinical settings and the application of integrative diagnostic reasoning in clinical practice. This gap—between mastering ultrasound technique and applying ultrasound findings within diagnostic reasoning and patient care—is the central and most educationally significant contribution of this study, and it frames the interpretation offered throughout this section.

### The Duality of Competency: Confidence versus Capability

A central tension emerged between students’ self-reported confidence and faculty-observed capability. Students described high confidence in foundational “knobology” and image acquisition on standardized patients, consistent with literature demonstrating improved motivation and perceived preparedness.^8^,^11^ Faculty corroborated this technical proficiency, with most noting strong probe placement skills.

However, technical mastery did not consistently translate into clinical sonographic competence—the ability to select, perform, interpret, and integrate ultrasound to answer a clinical question.^5^ As highlighted by faculty, students could obtain standard views but struggled to synthesize pathological findings. Students themselves acknowledged this gap, describing their ability to “see” without yet being able to “decide”.

This finding aligns with Miller’s Pyramid,^26^ where training achieves the “shows how” level but does not consistently reach the “does” level. Within this context, progression from controlled performance to real-world application appears insufficiently scaffolded, leaving clinical competence to develop opportunistically.

Several interrelated educational factors likely explain this incomplete progression. First, the curriculum’s teaching and assessment activities are concentrated almost entirely within the preclinical years, providing intensive but time-limited exposure that is not reinforced through longitudinal contact once students enter clerkships; without repeated, spaced engagement, skills acquired early are prone to decay and are rarely re-contextualized against real clinical problems. Second, preclinical training relies heavily on standardized patients and phantoms with normal findings, so students accumulate substantial deliberate practice^27^ in image acquisition but comparatively little in image interpretation under uncertainty—the cognitive skill most central to clinical application. Third, the largely opportunistic nature of clinical-year exposure means that when students do encounter real pathology, they typically do so without the structured, expert-modelled reasoning that underpins cognitive apprenticeship;^28^ faculty are rarely positioned to consistently narrate their diagnostic reasoning process at the bedside, so students are left to infer clinical integration independently rather than having it made explicit. Together, these factors suggest that the observed gap is not simply a matter of insufficient practice time, but reflects a curricular structure that scaffolds technical performance well while leaving the transition to workplace-based clinical reasoning largely unsupported.

### The Clinical Clerkship: A Curricular Gap

The most significant systemic issue was the absence of structured ultrasound training during clinical rotations. All students reported that exposure became opportunistic and rotation-dependent, effectively transforming a mandatory preclinical skill into an optional activity. This variability introduces inequity and limits opportunities for deliberate practice, a key determinant of skill acquisition.^27^

Within this context, these findings challenge the effectiveness of a “front-loaded” ultrasound course model and support the need for longitudinal, spiral integration.^5^,^17^ The clinical clerkship should represent the phase in which pattern recognition (normal versus abnormal) and clinical integration are explicitly taught and assessed. Although peer-led practice emerged as a compensatory strategy, it cannot replace structured, expert-guided clinical learning.

Addressing this gap would require embedding ultrasound explicitly within workplace-based learning during clerkships, rather than treating clinical exposure as an informal supplement to preclinical training. This could include designated ultrasound objectives within each core rotation, supervised bedside scanning of patients with real pathology, and structured debriefing in which supervising clinicians make their diagnostic reasoning explicit—operationalizing the cognitive apprenticeship model^28^ at the point of care. Such an approach would also create the repeated, feedback-rich exposure that deliberate practice requires,^27^ addressing the gap identified in The Duality of Competency: Confidence versus Capability between abundant preclinical practice in image acquisition and limited clinical-year practice in image interpretation and integration.

### Positioning the Findings Within the Broader Literature

Prior studies have documented positive learner perceptions, gains in basic ultrasound skills, benefits of focused educational interventions, and implementation of undergraduate ultrasound curricula across a range of settings.^19^,^20^,^22–25^ These studies differ substantially in design and scope, and many focus on a particular intervention, learner outcome, or curricular component. The present study therefore does not claim that the technical–clinical disconnect is entirely novel. Rather, its contribution is to characterize this disconnect from two stakeholder perspectives within the same undergraduate program and to identify curricular mechanisms that may help explain why it persists.

The convergence of independently collected student and faculty accounts strengthens the credibility of the finding: both groups described a strong technical foundation but limited progression toward interpretation of pathology and integration with clinical decision-making. Longitudinal undergraduate ultrasound curricula have been described internationally,^14–17^ whereas published evidence from the MENA region remains comparatively limited.^18^,^19^ Within this context, the present study adds a regional, stakeholder-informed perspective and highlights three potentially important mechanisms: concentrated preclinical exposure, an imbalance between acquisition and interpretation practice, and limited explicit modeling of ultrasound-based clinical reasoning during clerkships. These findings help move the discussion from whether a gap exists toward how curriculum design may contribute to it and how that transition could be better supported.

### Synthesis of Stakeholder Recommendations

Participants in this study (students and faculty) converged on three key pillars for curriculum development:

#### Longitudinal and Spiral Integration

Both groups emphasized the need to extend ultrasound training across all years of the curriculum. A spiral model would progressively build competence from foundational recognition in preclinical years to clinical application and diagnostic reasoning during clerkships.

#### Pathological Exposure and Clinical Reasoning

A major limitation identified was the focus on normal anatomy. Students noted limited exposure to pathology, while faculty confirmed deficits in interpreting abnormal findings. Structured exposure to pathological cases—through simulation, curated patient encounters, and case-based teaching—is essential. This aligns with cognitive apprenticeship principles, where expert reasoning is made explicit to learners.^28^

#### Standardized Competencies and Workplace-Based Assessment

Embedding ultrasound competencies within core clerkships was strongly advocated. Assessment should extend beyond OSCEs to include workplace-based tools such as Direct Observation of Procedural Skills (DOPS), enabling evaluation of integrated performance in authentic clinical settings.^29^

## Strengths and Limitations

### Strengths

The dual-perspective design enhances analytical depth and mitigates single-stakeholder bias. The triangulated analytic approach, combining manual and AI-assisted coding with consensus, strengthens credibility and confirmability. Adherence to COREQ guidelines and inclusion of reflexivity further support methodological rigor.

### Limitations

This study was conducted at a single institution in Qatar, which may limit transferability to other educational contexts. Recruitment relied on convenience sampling: participants self-selected in response to an open invitation, and the modest student response rate (21 of 69 eligible students, 30%) means that those who volunteered may not be representative of the full cohort—for example, students with stronger interest in, or more positive experiences of, ultrasound training may have been more likely to participate, potentially skewing the findings toward more favourable (or, conversely, more critical) accounts. Faculty participation was higher (15 of 19 eligible, 79%), which somewhat mitigates this concern for the faculty dataset. Purposive or stratified sampling in future work could help address this limitation. The inclusion of only emergency medicine faculty excludes perspectives from other specialties. The cross-sectional design precludes assessment of long-term skill retention and clinical impact. Although carefully managed, the use of AI-assisted coding represents a novel methodological approach, and while all outputs were independently verified against source transcripts by two researchers, the use of a proprietary, non-deterministic language model means that exact reproducibility of the preliminary AI-generated codes cannot be guaranteed. Variability in interview duration may have influenced depth of data; however, thematic saturation was achieved.

## Future Directions

Future research should focus on the implementation and evaluation of longitudinal ultrasound curricula within similar educational settings. This includes the development of standardized clerkship objectives, faculty training, and robust assessment frameworks, alongside prospective evaluation of skill retention, utilization, and diagnostic accuracy.

## Conclusion

This study provides a context-specific framework for advancing undergraduate ultrasound education. By integrating student and faculty perspectives, it highlights key competency gaps and system-level barriers. Its central contribution is the clear articulation of a translational gap between mastering ultrasound technique and developing the clinical reasoning required to apply ultrasound findings in diagnostic and patient-care decisions—a distinction that should anchor future curriculum reform. In this setting, ultrasound education should not remain confined to preclinical training as a standalone technical skill. Instead, it should be developed as a longitudinal, clinically integrated component of the curriculum, with explicit attention to supervised clinical application, workplace-based learning, and the deliberate integration of ultrasound into clinical reasoning activities during clerkships.

For educators in Qatar and similar educational contexts, these findings offer a stakeholder-informed rationale for transitioning from introductory ultrasound teaching toward structured, clinically embedded training models.

## Data Availability

Approved by Qatar University Institutional Review Board (QU-IRB 334/2024-EA). Written informed consent was obtained from all participants, including consent for publication of anonymized quotes. De-identified data are available from the corresponding author upon reasonable request.
